# Extensions to Regret-based Decision Curve Analysis: An application to hospice referral for terminal patients

**DOI:** 10.1186/1472-6947-11-77

**Published:** 2011-12-23

**Authors:** Athanasios Tsalatsanis, Laura E Barnes, Iztok Hozo, Benjamin Djulbegovic

**Affiliations:** 1 Center for Evidence-based Medicine and Health Outcomes Research, University of South Florida, Tampa, FL, USA; 2 Department of Mathematics, Indiana University Northwest, Gary, IN, USA; 3 H. Lee Moffitt Cancer Center & Research Institute, Tampa, FL, USA

## Abstract

**Background:**

Despite the well documented advantages of hospice care, most terminally ill patients do not reap the maximum benefit from hospice services, with the majority of them receiving hospice care either prematurely or delayed. Decision systems to improve the hospice referral process are sorely needed.

**Methods:**

We present a novel theoretical framework that is based on well-established methodologies of prognostication and decision analysis to assist with the hospice referral process for terminally ill patients. We linked the SUPPORT statistical model, widely regarded as one of the most accurate models for prognostication of terminally ill patients, with the recently developed *regret based decision curve analysis *(*regret DCA*). We extend the *regret DCA *methodology to consider harms associated with the prognostication test as well as harms and effects of the management strategies. In order to enable patients and physicians in making these complex decisions in real-time, we developed an easily accessible web-based decision support system available at the point of care.

**Results:**

The web-based decision support system facilitates the hospice referral process in three steps. First, the patient or surrogate is interviewed to elicit his/her personal preferences regarding the continuation of life-sustaining treatment vs. palliative care. Then, *regret DCA *is employed to identify the best strategy for the particular patient in terms of threshold probability at which he/she is indifferent between continuation of treatment and of hospice referral. Finally, if necessary, the probabilities of survival and death for the particular patient are computed based on the SUPPORT prognostication model and contrasted with the patient's threshold probability. The web-based design of the CDSS enables patients, physicians, and family members to participate in the decision process from anywhere internet access is available.

**Conclusions:**

We present a theoretical framework to facilitate the hospice referral process. Further rigorous clinical evaluation including testing in a prospective randomized controlled trial is required and planned.

## Background

### Introduction

Hospice services have been proven to provide better quality of care to dying patients[1-3] by optimizing pain relief [4,5] and reducing emotional stress [1,6,7]. Furthermore, hospice care is associated with greater patient-family satisfaction[8], is shown to be cost effective[9,10], and most importantly, it has been attributed with increased survival in some patients [11]. Despite these well documented advantages, many terminally ill patients do not reap maximum benefits from hospice care. The fundamental reason for this is related to the **less than optimal and frequently poorly timed referral **of terminally ill patients to hospice [1,12]. As a result, many patients die within a few days of referral, or live many years after the referral was made [13].

According to Medicare regulations, a person should be referred to hospice if his/her "life expectancy (LE) is 6 months or less" [1,14]. Hence, the problem of meaningful referrals relates to the accurate estimation (prognosis) of death within approximately 6 months after evaluation for hospice care. However, statistical models designed to assist physicians in predicting life expectancy (LE), although beneficial [15,16], so far they failed to improve the quality of care at the end of life [17-21].

One such statistical model is SUPPORT (Study to Understand Prognoses and Preferences for Outcomes and Risks of Treatments), designed to calculate the probability of survival over a period of 180 days [22,23]. Although the SUPPORT model has been well validated [17,22] for prognostication of LE in terminally ill patients, a controlled trial of SUPPORT failed to demonstrate any impact on the overall quality of care for these patients [17,20]. We postulate that this lack of impact may be due to the fact that SUPPORT results, were not linked to any decision methodology that would translate the probability of survival to a hospice referral recommendation. Therefore, the full potential of the model's prognostication power remained unexploited.

In this work, we link the SUPPORT prognostication model with the recently developed decision methodology *regret DCA *[24] to facilitate the hospice referral process. *Regret DCA *relies on regret theory and decision curve analysis [25] to recommend the optimal management strategy for a patient, accounting for the personal attitudes and values of the particular patient or his/her surrogate.

Furthermore, we extend *regret DCA *to incorporate harms and effects of treatment as well as harms associated with the prognostication test to the decision model. The presented methodology is integrated into a comprehensive clinical decision support system developed to facilitate the hospice referral process.

## Methods

### Dataset

In our analysis, we utilized the entire SUPPORT dataset, both development and validation cohorts. The dataset is presented in detail elsewhere [22]. Medical records of 8,329 seriously ill hospitalized adults are included.

### Support model

SUPPORT is a multivariable model designed to estimate probability of survival for seriously ill hospitalized patients over a period of the subsequent 180 days. The model variables include the patient's medical condition compatible with one of eight major diagnostic groupings (Acute Respiratory Failure, Multiple Organ System Failure, Chronic Obstructive Pulmonary Disease, Congestive Heart Failure, Hepatic Cirrhosis, Neurological Coma, Lung or Colon Cancer), the patient's current age, number of days in the hospital before study entry, neurologic status, and 11 physiologic measures recorded on day 3 after study entry [22].

The SUPPORT implementation for the estimation of survival probability is detailed in the appendix. Due to the nature of the hospice referral problem we also express the survival probability in terms of mortality. We can convert the estimated survival probability (SP) (equation A2) to probability of death within 180 days (denoted here as *p*) using the equation:

(1) p=1-SP=1-P{T≥t|disease group=i}

where *SP *is the survival probability computed by SUPPORT, *i *∈ [1,8] the patient's disease group, *T *is the survival time in days, and *t *is an arbitrary time (typically expressed in days e.g. *t *∈ [1,180]).

In terms of accuracy, the SUPPORT model has an area under the receiver-operating characteristics curve (ROC) for prediction of surviving 180 days of 0.79 in the phase I development cohort and 0.78 in the phase II validation cohort [22].

### Decision model

**Figure 1:**
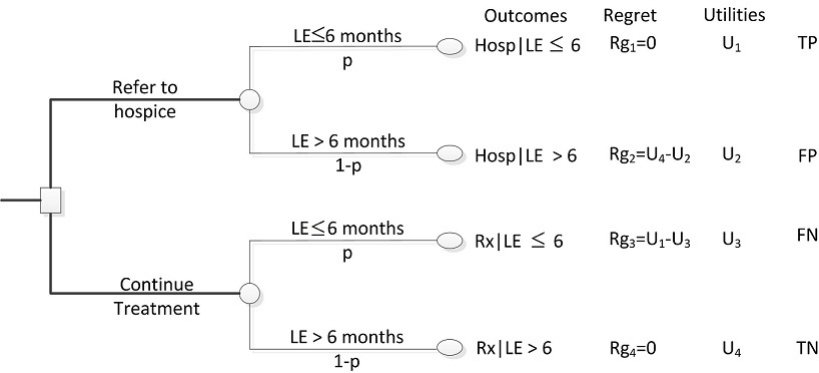
**Decision tree for hospice referral**. In this figure, p is the probability that a patient's LE is less than or equal to 6 months; 1-p is the probability that a patient's LE is greater than 6 months; U_i_: are the utilities associated with each outcome; Rg_i _is the regret associated with each outcome.

Figure 1 depicts the decision tree summarizing the process of hospice referral. The four outcomes and their corresponding utilities (*U*) shown are:

1. *U*_1_: Refer the patient to hospice and the patient's LE is less than or equal to 6 months (*Hosp*|*LE *≤ 6).

2. *U*_2_: Refer the patient to hospice and the patient's LE is greater than 6 months (*Hosp*|*LE *> 6).

3. *U*_3_: Continue treating the patient and the patient's LE is less than or equal to 6 months (*Rx*|*LE *≤ 6).

4. *U*_4_: Continue treating the patient and the patient's LE is greater than 6 months (*Rx*|*LE *> 6).

*p *is the probability associated with the presence of an event (e.g. patient's LE ≤ 6 months) as predicted by the SUPPORT model, 1 - *p *is the probability associated with the absence of the same event (e.g. patient's LE > 6 months).

As with any decision, one may come to realize that, in retrospect, an alternative decision would have been preferable. This knowledge may bring a sense of loss or regret [26-32]. In this paper, we use this sense of regret to determine the preferences of the decision maker towards alternative management strategies. Specifically, we employ regret theory to estimate the threshold probability, *P_t_*, at which the decision maker (patient, physician, or family member) is indifferent between continuation of treatment vs. hospice referral. Based on the concept of threshold probability, the patient should be referred to hospice if his/her probability of death is greater than or equal to *P_t_*(e.g. *p *≥ *P_t_*), and he/she should continue receiving curative treatment otherwise (*p *<*P_t_*).

The threshold probability is derived as [24]:

(2) $$P_{t}=\frac{1}{1+\frac{U_{1}-U_{3}}{U_{4}-U_{2}}}$$

In (2) *U_1_-U_3 _*is associated with regret of omission (e.g. the patient was not referred to hospice, instead he/she continued receiving unnecessary treatment) and *U_4_-U_2 _*with regret of commission (e.g. the patient was unnecessary referred to hospice instead of continue receiving life-sustaining treatment) [24].

**Figure 2:**
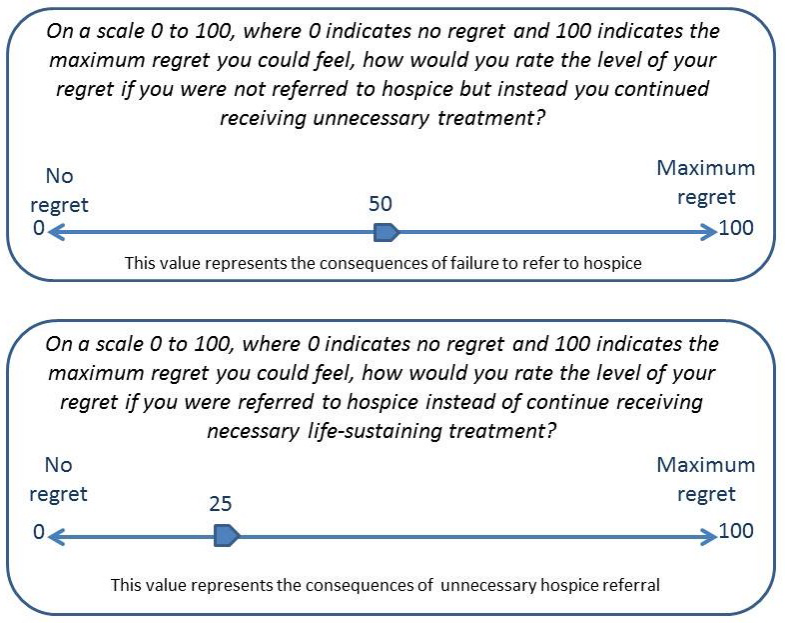
**DVAS (Dual Visual Analogue Scales)**. The DVAS are used for the elicitation of the decision maker's threshold probability.

To elicit the decision maker's regret, and therefore threshold probability, we utilize the DVAS (Dual Visual Analogue Scale) method [24]. One visual analogue scale is used to capture the regret associated with failing to refer the patient to hospice (e.g. continue unnecessary treatment) and the second scale to measure the regret associated with unnecessary hospice referral (e.g. failing to provide life-sustaining treatment) (Figure 2).

Elicitation of threshold probability can be achieved through a set of questions such as:

1. On the scale 0 to 100, where 0 indicates no regret and 100 indicates the maximum regret you could feel, how would you weigh the level of your regret if you were not referred to hospice but instead you continued receiving unnecessary treatment? That is, how much would you regret if you did not reap the benefits of hospice care? *Note that this value corresponds to U_1 _- U_3_.

2. On the scale 0 to 100, where 0 indicates no regret and 100 indicates the maximum regret you could feel, how would you weigh the level of your regret, if you were referred to hospice instead of continue receiving necessary life-sustaining treatment? That is, how much would you regret if you sustained harms from hospice care? *Note that this value corresponds to U_4 _- U_2_.

For example, suppose that the patient - who is aware of his/her terminal condition- answers 50 and 25 to the questions 1 and 2 respectively. This means that the patient considers 50/25 = 2 times worse not to be referred to hospice when necessary than receiving an unnecessary hospice referral. The threshold probability for this patient is (equation 2)

$$p_{t}=\frac{1}{1+\frac{U_{1}-U_{3}}{U_{4}-U_{2}}}=\frac{1}{1+\frac{50}{25}}=0.33\;\text{or}\;\text{33\% }\text{.}$$

### *Regret DCA *and extensions

The clinical problem we face in the situation of hospice referral is how to use reasonably accurate predictions of death, *p*, coupled with the patient's preferences (as expressed in terms of threshold probability, *P_t_*) to arrive at the optimal decision for a specific individual. The problem is decomposed into three strategies: (1) act based on the prediction model (SUPPORT) (e.g. refer to hospice if *p *≥ *P_t _*and continue treating otherwise), (2) refer all patients to hospice, and (3) continue current treatment for all patients (i.e. refer no patients to hospice).

Each of these strategies may inflict physiological and/or psychological damages to the patient. Specifically, a patient may suffer harms due to a treatment strategy (e.g. adverse effects) or harms due to the prognostication test (e.g. a test requiring invasive procedure). We express these harms as loss in utility associated with actions we may undertake. To that end, we define *H_Rx_*, *H_Hosp _*and *H_te _*as the utility losses due to harms of the treatment, hospice, and prognostic test, respectively.

**Figure 3:**
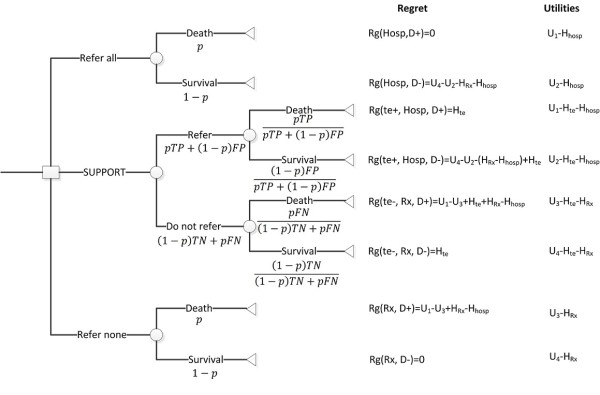
**Decision tree describing the overall hospice referral process**. In this figure p = P (D +): probability the patient's LE is less than or equal to 6 months; 1 - p = P (D -): probability the patient's LE is greater than 6 months; U_i_, i ∈ [1,4]: the utilities corresponding to each of the decision model outcomes; Hosp: hospice referral; Rx treatment continuation; Rg: regret associated with an action; H_Rx _: utility losses due to harms of treatment; H_Hosp_: utility losses due to harms of hospice; H_te_: utility losses due to harms of the prognostic test (SUPPORT).

Figure 3 presents the decision tree describing the overall hospice referral problem. *p *= *P*(*D *+) is the probability that the patient's LE is less than or equal to 6 months as estimated by the prediction model (SUPPORT);1 - *p *= *P*(*D *-) is the probability that the patient's LE is greater than 6 months, and *U_i_*, *i *∈ [1,4], are the utilities corresponding to each of the decision model outcomes (detailed in the previous section). The variables *Hosp *and *Rx *correspond to referring a patient to hospice and continuing current curative treatment, respectively. *Rg*, is the regret associated with an action, e.g. *Rg*(*Hosp*, *D*-) is the regret one may feel if the patient was referred to hospice when his/her LE was greater than 6 months. Finally, *te *designates that the patient received a prognostication test.

Considering the decision tree in Figure 3 we can compute the expected regret associated with each decision in terms of the utilities of each possible outcome as follows (detailed derivation is presented in the Appendix):

(3) $$ERg\left[Hosp\right]=\left(1-p\right)*\left(1-RRR_{Hosp}\right)*\frac{P_{t}}{1-P_{t}}$$

(4) $$ERg\left[Rx\right]=p*\left(1-RRR_{Rx}\right)$$

(5) $$\begin{array}{c} ERg\left[SUPPORT\right]=\\ \left(1-RRR_{Hosp}*\left(\#TP∕n+\#FP∕n\right)\right)\\ \left(-RRR_{Rx}*\left(\#FN∕n+\#TN∕n\right)\right)\\ {}*\frac{H_{te}}{U_{1}-U_{3}+H_{Rx}-H_{Hosp}}\\ +\left(1-RRR_{Hosp}\right)*\frac{\#FP}{n}*\frac{P_{t}}{1-P_{t}}\\ +\left(1-RRR_{Rx}\right)*\frac{\#FN}{n} \end{array}$$

In addition to harms, equations 3, 4 and 5 incorporate the effects of treatment and hospice care using measures of *Relative Risk Reduction: *and *RRR_Rx _RRR_Hosp _*respectively. The values for these measures are treatment specific and can be acquired from the literature. We have incorporated hospice effects because a recent study [11] has shown that early palliative care for patients with metastatic non-small cell lung cancer could increase survival. The variables *TP*, *FP*, *FN*, *TN *are related to the prognostic capability of the SUPPORT model (see appendix for detailed derivation) [24].

Since the regret of omission and regret of commission have been generalized to include effects and harms related to management strategies and testing, the function of threshold probability (equation 2) becomes:

(6) $$P_{t}=\frac{1}{1+\frac{U_{1}-U_{3}+H_{Rx}-H_{Hosp}}{U_{4}-U_{2}-H_{Rx}-H_{Hosp}}}$$

Where *U*_1 _- *U*_3 _+ *H_Rx _*-*H_Hosp _*corresponds to the regret associated with not referring the patient to hospice when necessary, and *U*_4 _- *U*_2 _- *H_Rx _*- *H_Hosp _*corresponds to the regret associated with unnecessary hospice referral.

### Choosing the optimal strategy

The optimal strategy is selected as the one which will bring the least amount of regret. The *regret DCA *algorithm expresses the regret associated with each strategy in terms of threshold probability and is implemented as follows [24]:

1. Select a value for threshold probability.

2. Assuming that patients should be referred to hospice if *p *≥ *P_t _*and should continue current treatment otherwise, compute #TP and #FP for the prediction model.

3. Calculate the *ERg*(*SUPPORT*)using equation 5.

4. Calculate *ERg*(*Rx*) using equation 4.

5. Compute the *ERg*(*Hosp*)using equation 3.

6. Repeat steps 1 - 6 for a range of threshold probabilities.

7. Graph each expected regret function calculated in steps 3-5 against each threshold probability.

**Figure 4:**
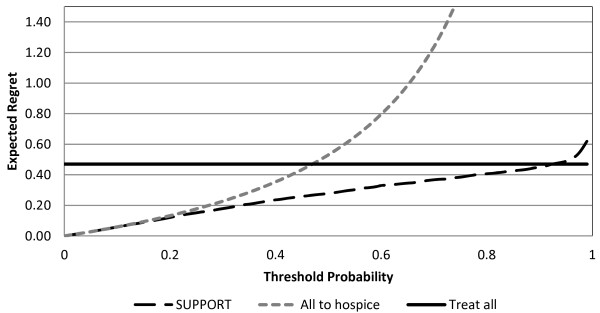
**Decision curves for hospice referral**. In this figure, RRR_Hosp _= 0, RRR_Rx _= 0, H_Rx _= H_Hosp _= H_te _= 0. At threshold probability equal to 10%, the optimal decision is refer the patient to hospice; at 40% the optimal decision is to use the SUPPORT model.

At each threshold probability, **the action with the lowest value of expected regret corresponds to the most desired action**. For example, in Figure 4, at a threshold probability equal to 10% (e.g. the patient considers 9 times worse not to be referred to hospice when necessary than to receive an unnecessary hospice referral), the optimal strategy is to refer the patient to hospice.

**Figure 5:**
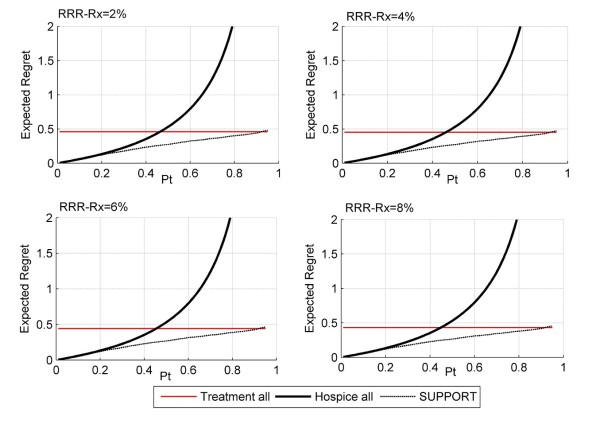
**Decision curves as a function of RRR_RX_**. In this figure, RRR_Hosp _= 0, RRR_Rx _= 2 to 8%, H_Rx _= H_Hosp _= H_te _= 0. As the effect of treatment increases, the regret associated with treating all patients and with the SUPPORT model slightly decreases. The strategy of using the SUPPORT model to refer a patient to hospice is the action with the least amount of regret for the wider range of threshold probabilities.

**Figure 6:**
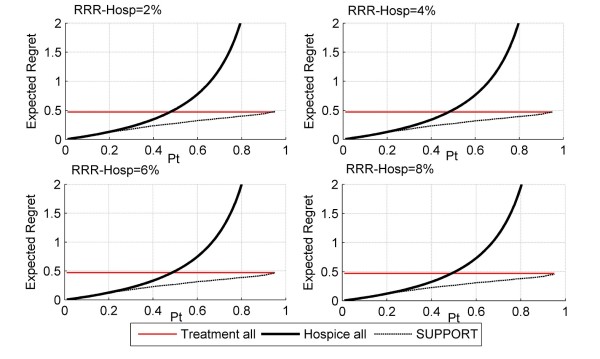
**Decision curves as a function of RRR_Hosp_**. In this figure, RRR_Hosp _= 2% to 8%, RRR_Rx _= 0, H_Rx _= H_Hosp _= H_te _= 0. As the effect of hospice care increases, the regret associated with hospice and with the SUPPORT model slightly decreases. As previously, the strategy of using the SUPPORT model to refer a patient to hospice is the action with the least amount of regret for a wide range of threshold probabilities.

**Figure 7:**
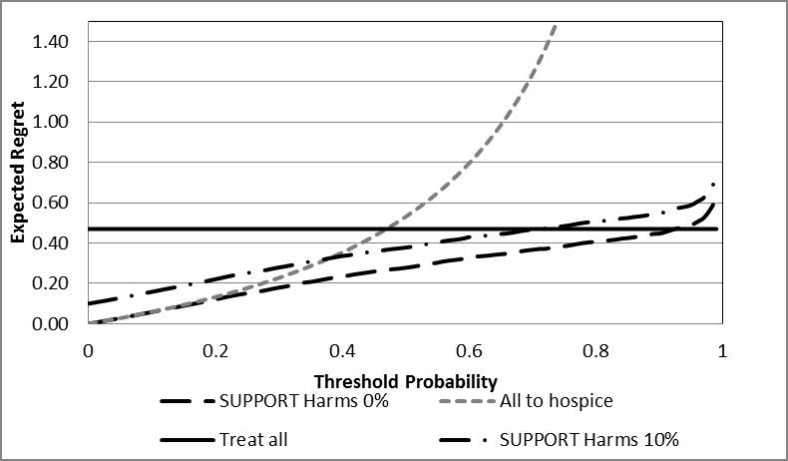
**Decision curves as a function of H_te_**. In this figure, RRR_Hosp _= 0, RRR_Rx _= 0, H_Rx _= H_Hosp _= 0 and H_te _= 0% and 10%. As the harms associated to the prediction test increase, so does the expected regret of utilizing the SUPPORT model for hospice referral. Increasing the harms due to treatment or due to hospice care does not have an effect on the decision curves.

Figures 5, 6 and 7 depict the regret associated with alternative decision strategies as they relate to different values of hospice effectiveness (Figure 5), treatment effectiveness (Figure 6), and harms due to the prognostication test (Figure 7). As expected, when the harms due to the prognostication test are increased, then the area of threshold probability at which the prognostication model is the optimal decision is reduced (Figure 5). Even though, it is not expected that the SUPPORT model will actually create harms, at least physiological, to the patient, this is not always the case for other diagnostic tests that may be more invasive (e.g. screening for prostate cancer).

As can be seen from Figures 5, 6 and 7 the optimal decision is derived by the SUPPORT model for a rather wide range of threshold probabilities. Therefore, it appears that the SUPPORT model is the superior strategy for the vast majority of decision makers, regardless the effects of the alternative management strategies. However, since the threshold probability expresses the personal preferences of a particular decision maker, it is not unusual for specific patients to have smaller or greater threshold probability values than the majority of decision makers. This is the power of the proposed methodology, which allows for decision making at the individual level. For example, if the decision maker presents a threshold probability greater than ≈92%, the optimal decision would be to continue life-sustaining treatment even if it is deemed not to be effective(Figure 4). Similarly, for small values of threshold probability, the desired action would be to refer the patient to hospice.

### Decision Support System

As our theoretical discussion highlighted, decisions about life and death are complex and difficult at both the emotional and cognitive level. Therefore, it is not surprising that the SUPPORT model originally failed to improve the quality of care for terminally ill patients despite its reasonable accuracy in prediction of probability of survival [17,20]. Any attempt to focus on a single dimension of the complex hospice referral process is not likely to succeed. An accurate prognostic model is only the first step. Having the apparatus to take into account trade-offs associated with the hospice referral decision while taking into consideration the patients' preferences represent further necessary steps to improve the care of terminally ill patients. In addition, we hypothesize that the SUPPORT intervention failed because it was not available at the point of care in real time. This is because the most desired outcomes are best achieved when decision-making occurs in real-time, at the point of care [33,34].

To facilitate the decision making process for the hospice referral at bedside, we propose a web-based clinical decision support system (CDSS) that computes the probabilities of survival and death for individual patients using the SUPPORT model, elicits personal preferences from patients and/or physicians, and utilizes *regret DCA *to suggest the optimal decision for a particular patient.

### Features

#### Access

Our goal is to develop a CDSS that can be accessed by everyone and from anywhere regardless the operating system one uses. At the same time, it is desirable to develop a system that can eventually be integrated with various healthcare providers' electronic medical records (EMR). We concluded that a web-based implementation would fulfil such requirements.

#### Data storage

The CDSS performs the required computations without retaining or transmitting sensitive and identifiable information.

## Results

**Figure 8:**
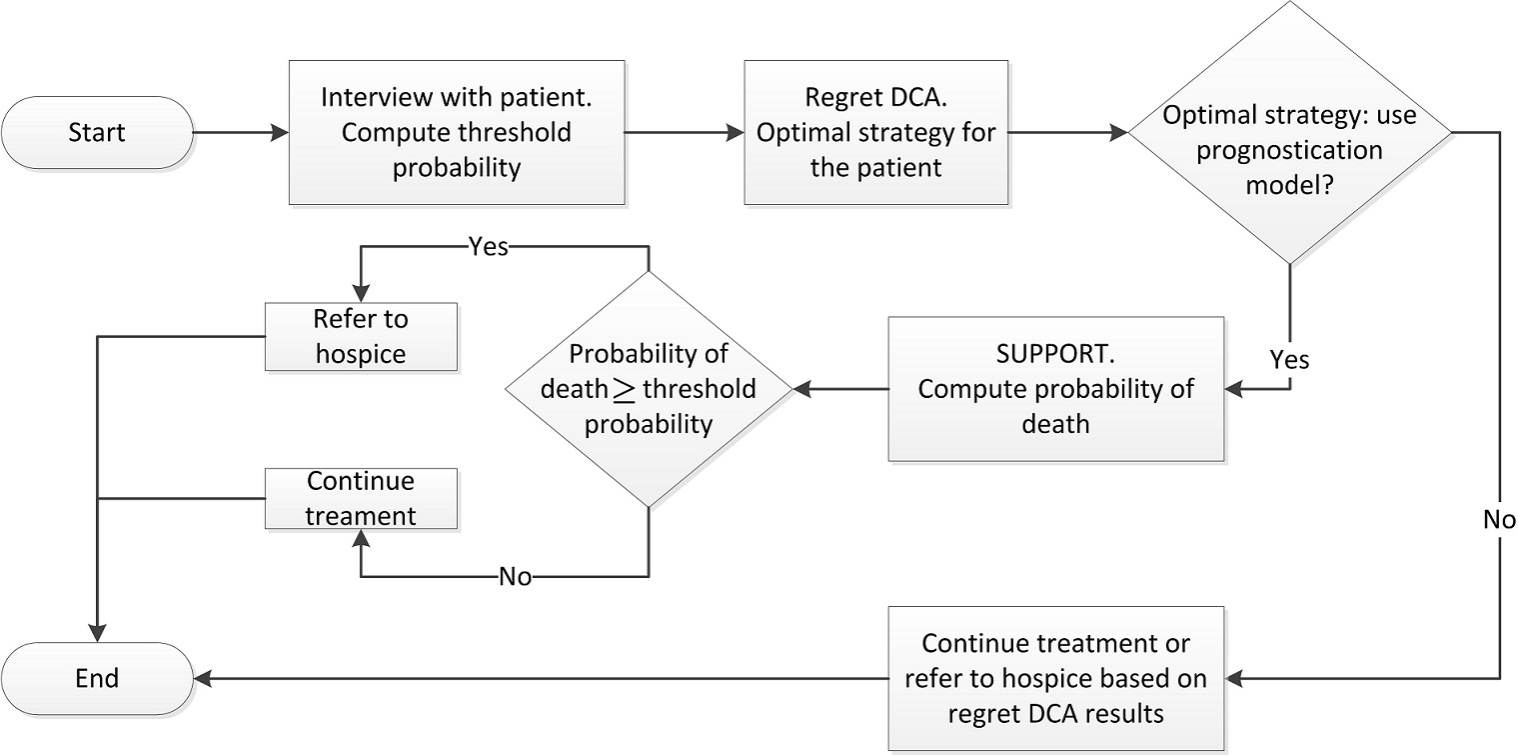
**Block diagram outlining the operation of the DSS**.

In this section we present a prototype of the CDSS, developed to demonstrate the applicability of our theoretical framework for hospice referral. Each subsection describes the results of the methods shown in the previous section in conjunction with the description of the corresponding module. Figure 8 depicts the logical diagram that outlines the operation of the CDSS. Briefly, the operation begins by interviewing the patient or surrogate to elicit his/her threshold probability. Based on the value of threshold probability equation, the optimal strategy for the particular patient is derived (e.g. refer to hospice, continue treatment, or use of prediction model). If the optimal strategy is to follow the prediction model (SUPPORT), then using the equations A1, A2 and 1, the probabilities of survival and death are computed for the particular patient. The probability of death is then contrasted with the patient's threshold probability and the optimal decision is derived (refer to hospice, or continue treatment). At each step described, the patient selects the level of information he/she wishes to be exposed to. For example, the patient may not wish to know his/her threshold probability or probability of death. Instead he/she wishes to know only the optimal decision regarding his/her condition.

### General implementation details

The proposed CDSS is a web-based application residing on the USF Health servers. The web address is http://health.usf.edu/research/ebm/decisionaids.htm. It has been developed based on the Adobe^® ^ColdFusion^® ^application technology and the interface has been designed using html and JavaScript programming languages. The hardware and software requirements from the user's point of view are modest. The system runs on any contemporary computer with net browsing capabilities. However, at this stage the CDSS is not optimized for use with handheld devices. The CDSS consists of 3 different modules as described below.

### Elicitation of threshold probability module

**Figure 9:**
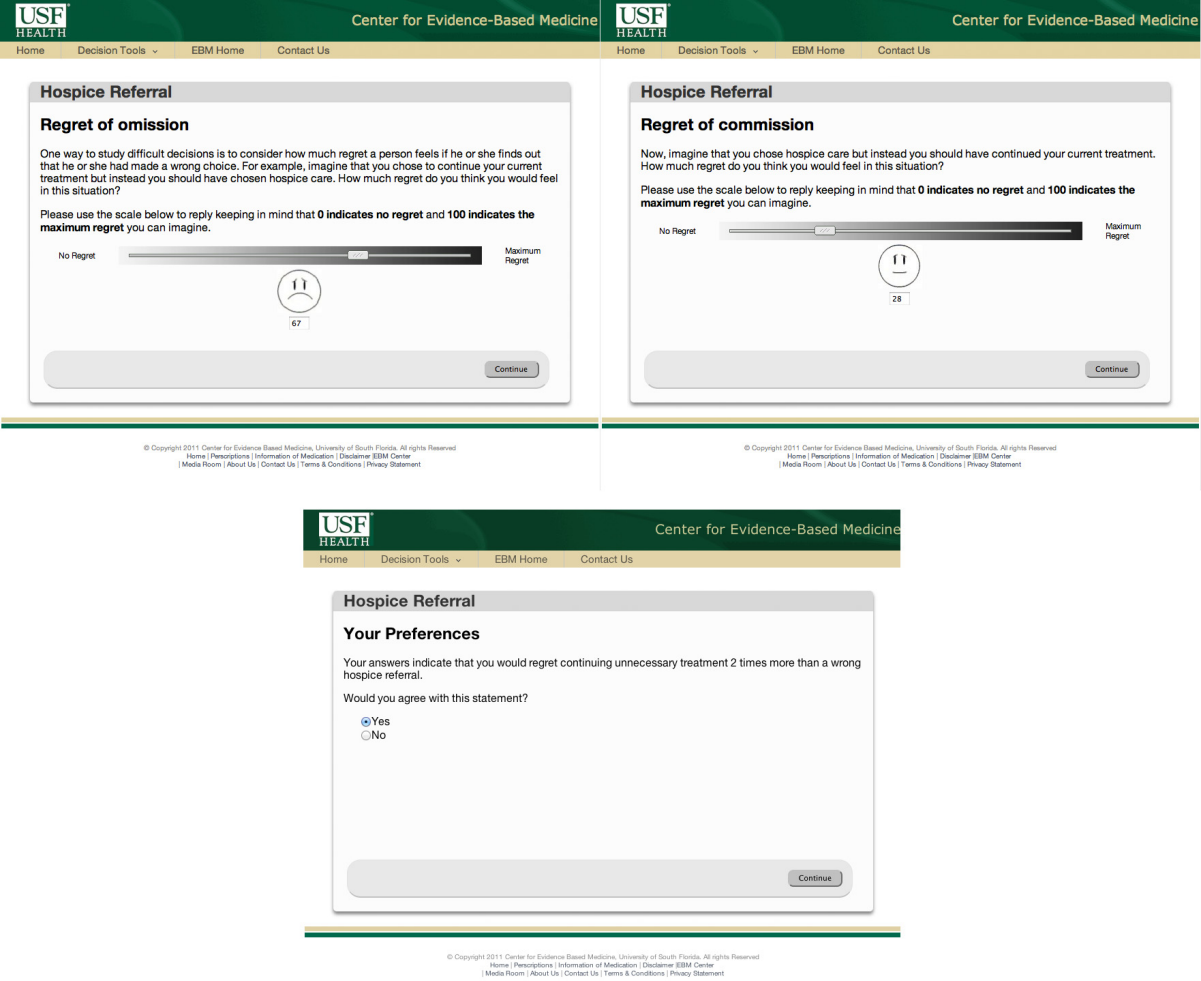
**Elicitation of threshold probability**. The user (patient/surrogate/physician) weighs the two alternative management strategies in terms of regret.

The threshold elicitation module consists of the dual visual analogue scales, used to weigh the patient's regret in the case of wrong decisions. Each scale has 100 points where 0 corresponds to no regret and 100 to maximum regret. Depending on the role of the decision maker (e.g. patient/surrogate or physician) two different sets of questions are displayed. These questions are designed to capture the regret of omission and the regret of commission. For the remainder of this paper, we assume that the decision maker is the patient. As in pain scales [35], each visual analogue scale uses facial expressions to graphically represent variations in regret (Figure 9). A summary of the decision maker's preferences is presented for final verification. The threshold probability for the particular patient is derived using equation 2, however is not displayed until the decision maker requests it.

### Decision module

The decision module utilizes the decision maker's threshold probability and the regret DCA methodology to derive the optimal decision. For example, the preferences of the patient depicted in Figure 9, correspond to a threshold probability equal to 29%. From Figure 4 the strategy that will bring the least amount of regret is to use the prognostication model (SUPPORT) for the hospice referral recommendation. In this case, the decision module initiates the SUPPORT module.

### SUPPORT module

**Figure 10:**
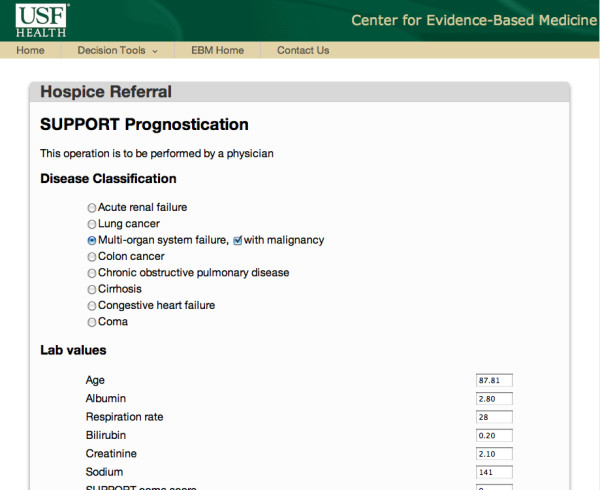
**SUPPORT user interface**. The user enters all information regarding the particular patient to compute the probability of death and survival within the next 6 months. LE results are presented to the patient through the decision justification module after the patient's request.

If the optimal strategy derived by the decision module is to utilize the prognostication model, the SUPPORT module is enabled. This module (Figure 10) is used to compute the probability of death for the particular patient based on the SUPPORT prognostication model. Currently, the user inserts all required information to the CDSS. In the future, this information will be captured automatically from the health care provider's electronic medical records system. Data validation restrictions have been imposed to protect the integrity of the collected data.

Once the values of all available variables have been inserted in the corresponding cells, the patient's life expectancy and probabilities of survival and death are computed. The decision module is employed again to display the optimal recommendation.

### Decision justification module

**Figure 11:**
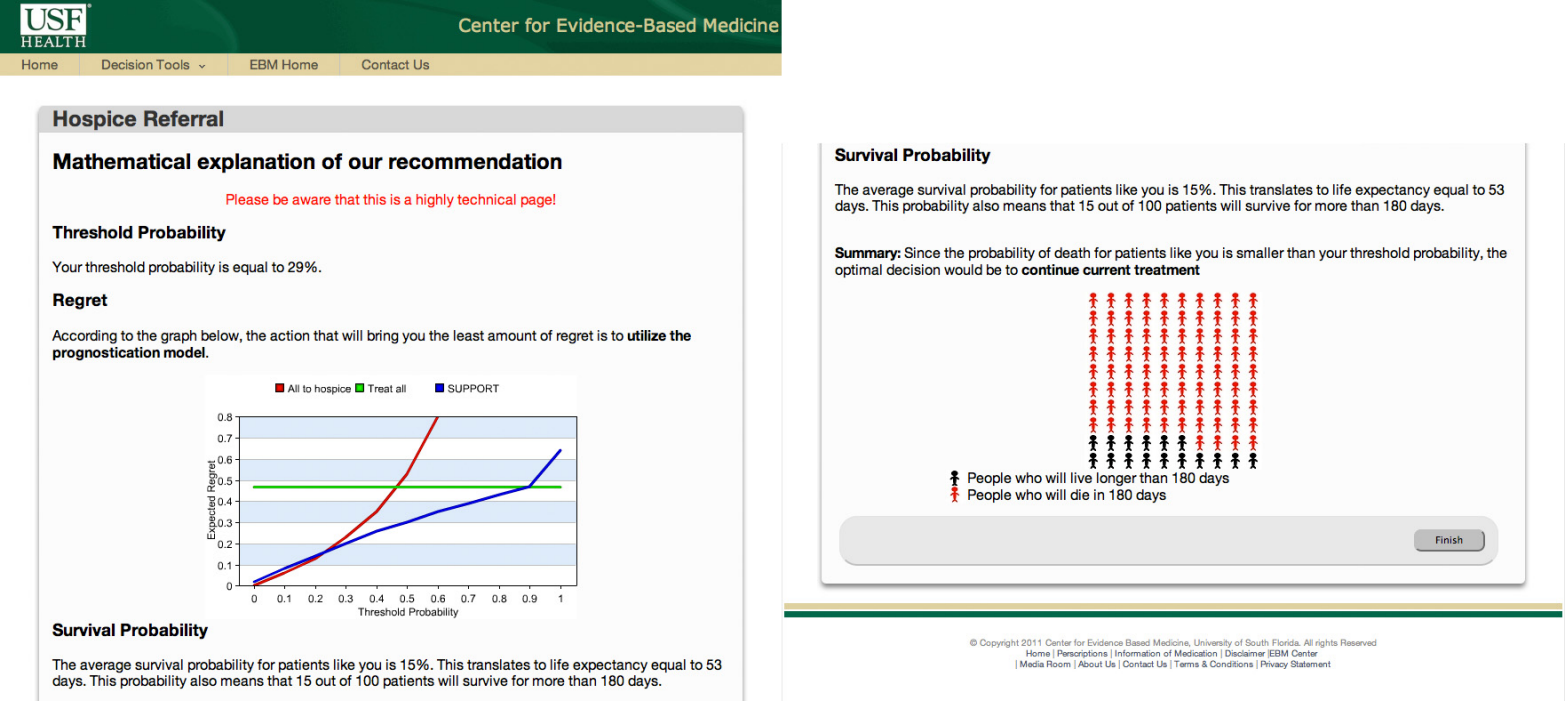
**Justification of the hospice referral recommendation**. The particular patient depicted has 29% threshold probability at which the optimal strategy is derived by the SUPPORT model. The patient has 85% probability of death in the next 180 days. Therefore, the optimal decision is to be referred to hopsice.

The decision justification module explains in detail and at the user's request the reasons that led to a particular recommendation (Figure 11). It contains information regarding the decision maker's threshold probability, the optimal strategy associated with the threshold probability and the patient's probability of death (if applicable). Since people often misinterpret probabilities [36], we complement the results presented in terms of probabilities using frequency format (Figure 11). The latter format is currently considered the best way to represent favorable and unfavorable facts regarding medical interventions [37]. The justification module is highly technical and should only be reviewed by decision makers who wish to know more about their or their patient's condition.

## Case Study

**Figure 12:**
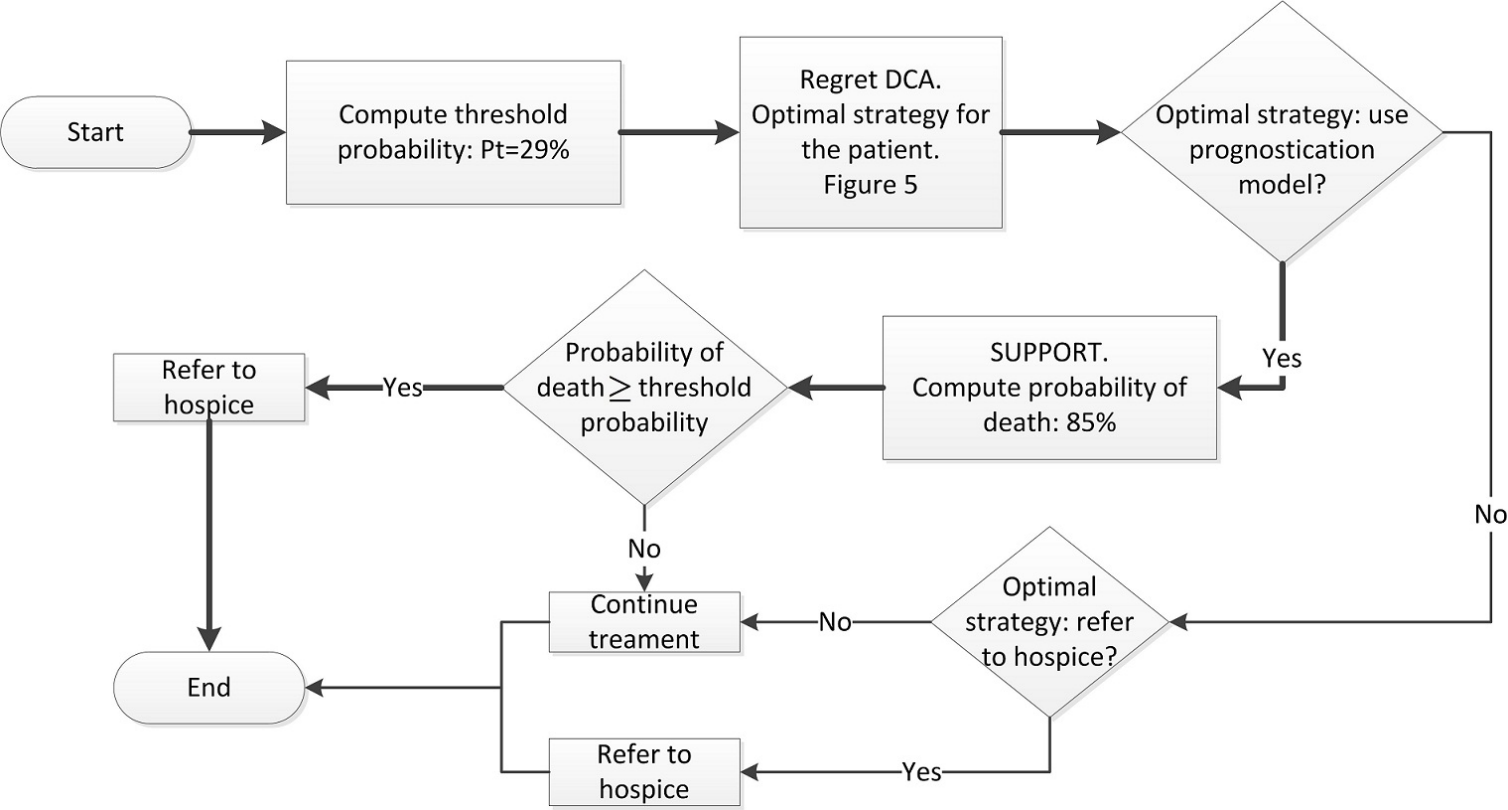
**Block diagram summarizing the decision process**. The bold route corresponds to the patient simulated in figures 10, 11 and 12.

Figure 12 summarizes the decision process for a patient whose information is simulated in Figures 10, 11 and 12. The probability of death and the threshold probability of this patient have been computed as 85% and 29% respectively. At a 29% threshold probability, the optimal strategy is to use the prediction model for hospice referral (Figure 4). Therefore, since *p *>*P_t _*the patient should be referred to hospice. For completeness, all possible decision routes are depicted in Figure 12. The route corresponding to the specific simulated patient is shown using bold arrows.

## Discussion

In this article we describe both the theory and application behind a hospice referral clinical decision support system. To the best of our knowledge, this is the first CDSS that integrates two well established methodologies, one for prognostication (SUPPORT) and the other for decision making (*regret DCA*), to assist with the hospice referral decision-making process.

The recently developed *regret DCA *incorporates the decision maker's preferences towards alternative management strategies from the perspective of regret theory in terms of threshold probability. Such an approach promotes personalized patient care. We anticipate that the regret-based approach is more appropriate for the hospice referral process than other preference elicitation techniques, due to the nature of the problem where there are really no optimal options available- the optimal decision can be only considered as the one with the least regret.

Modern cognitive theories increasingly focus on the so called dual-processing theory in which both intuition (system 1) and analytical, deliberative process (system 2) are important for balancing risks and benefits in the decision-making process [38]. We believe that rational decision-making should take into account both formal principles of rationality and human intuition about good decisions[24,39,40]. One way to accomplish this is to use regret, a cognitive emotion, to serve as the link between systems 1 and 2 [24]**. **By taking into account the consequences of our actions as well as the circumstances under which we can live with our mistakes we anticipate that the goal of reconciling the formal principles of rationality and human intuitions about good decisions can be met [24,29,39,40]. This is particularly true in the situation of terminally ill patients.

Our web-based CDSS reflects modern cognitive theories to facilitate integration of the decision-making ingredients necessary for hospice referral decisions. The CDSS encapsulates all required information for the hospice referral process into a flexible software that can be used at bedside. Obviously, hospice referral decisions are complex and must be exercised with full compassion and deliberation. We advise against the use of our system as an automatic decision making tool that by-passes important personal interactions between the patient and his/her physician. It is important to stress that the elicitation of the threshold probability as described herein reflects the belief (also captured in recent legislation [41]) that patients and their families want to be told the "truth" about the patients' terminal sickness [22,41,42] and that physicians have ethical obligations to share this information with patients and their families [41,42]. Our system should be understood as an aid to facilitate decisions in terminal phases of patient lives.

Our approach has limitations as well. The main limitation of the proposed system remains the complexity of the SUPPORT model. Currently, the system still requires manual entry of data. In addition, failure to enter all data can jeopardize the accuracy of prediction and therefore, the decision process. To cope with this limitation, we plan to integrate our system into various health providers' EMRs. Based on each EMR, specifically designed queries will be used to retrieve lab values and patient demographics to be fed automatically into our system; a process that will reduce the amount of missing values and input errors.

The second limitation of the proposed system is that empirical data are not available to assess how the system actually works in practice. While we plan to undertake empirical testing of the system described here, we believe that a strong theoretical underpinning will enable better hospice referral decisions even in the current form. This is because our system will essentially operationalize the decision-making process, which is supposed to occur in every day practice. Nevertheless, we need to firstly, identify the system's feasibility in real life settings and ultimately, if it appears to be usable and assessed favourably by all those involved in the hospice-referral decision-making process, to test it in randomized controlled trials against traditional care.

Our future plans include both empirical testing and implementation of multiple additional prognostication models which will be used in parallel to assess optimal decisions regarding hospice referral and take advantage of the regret DCA methodology. We anticipate that for a different range of threshold probabilities these models may perform better than the SUPPORT model. Furthermore, our intent is to develop a separate version of our CDSS optimized for mobile devices.

## Conclusions

In this work we have presented the theoretical framework, accompanied by the associated CDSS, to facilitate end of life care decisions. Our work combines the prognostication power of the SUPPORT model, the simplicity of the DVAS methodology in eliciting people's preferences and the effectiveness of regret DCA at evaluating alternative management strategies to resolve the dilemma of choosing traditional vs. palliative care for patients at terminal stages. A clinical evaluation of the CDSS is planned.

## Competing interests

The authors declare that they have no competing interests.

## Authors' contributions

All authors contributed equally to this work. All authors have read and approved the final manuscript.

## Appendix

### Support implementation

SUPPORT is implemented in two steps. First, the SUPPORT physiology score is computed based on equation A1 [22].

(A1) $$\begin{array}{l} SPS=\text{259}.\text{9\{}ARF\;/\;MOSF\text{\}}+\text{263}.\text{4\{}COPD\;/\;CHF\text{\}}\\ +\;\text{241}.\text{4\{}Cirrhosis\;/\;Coma\text{\}}\\ +\;\text{281}.\text{5\{}Lung\;/\;ColonCancer\text{\}}\\ -\;0.0\text{6174min}(PaO_{\text{2}}\;/\;FiO_{\text{2}},\text{225})\\ -\;0.\text{6316min(}MeanBP,\text{6}0\text{)}\\ +\;\text{1}.0\text{2}0\text{5}WBC-0.\text{3676(}WBC-{\text{8)}}_{+}\\ -\;0.\text{5631(}WBC-{\text{11)}}_{+}+0.\text{2691min(}Alb,\text{4}.\text{6)}\\ +\;0.\text{2312}Aresp-\text{2}.\text{362}Temp+\text{1}.\text{326(}Temp-\text{36}.{\text{6)}}_{+}\\ +\;\text{2}.\text{473(}Temp-\text{38}.{\text{3)}}_{+}-\text{1}.\text{579}\times \text{1}0^{–}{}^{\text{1}}HR\\ +\;\text{9}.\text{77}0\times \text{1}0^{–\text{5}}{(HR-\text{55)}}_{+}^{3}-\text{2}.\text{189}\times \text{1}0^{-\text{4}}{\left(HR-\text{8}0\right)}_{+}^{3}\\ +\;\text{1}.\text{518}\times \text{1}0^{-\text{4}}{\left(HR-\text{11}0\right)}_{+}^{3}\\ -\;\text{3}.0\text{62}\times \text{1}0^{-}{}^{5}{\left(HR-\text{149}\right)}_{+}^{3}+0.\text{9763}Bil\\ -\;0.\text{7481(}Bil-{\text{7)}}_{+}-\text{6}.\text{8761}Cr\\ +\;\text{11}.\text{6}0\text{58(}Cr-0.\text{6}{00\text{)}}_{+}^{3}-\text{21}.\text{8413(}Cr-{\text{1}.000\text{)}}_{+}^{3}\\ +\;\text{1}0.\text{3574}{\left(Cr-\text{1}.\text{5}00\right)}_{+}^{3}-0.\text{1219}{\left(Cr-\text{5}.\text{399}\right)}_{+}^{3}\\ -\;0.\text{6167}0\text{96}Na+0.00\text{21118(}Na-{\text{128)}}_{+}^{3}\\ -\;0.00\text{3673}0{\left(Na-\text{135}\right)}_{+}^{3}+0.000\text{6126(}Na-{\text{139)}}_{+}^{3}\\ +\;0.000\text{9486}{\left(Na-\text{148}\right)}_{+}^{3}\\ -\;\text{6}.\text{278\{}COPD\;/\;CHF\text{\}}\times \text{min(}Alb,\text{4}.\text{6)}\\ -\;\text{11}.\text{45\{}Lung\;/\;ColonCancer\text{\}}\times \text{min(}Alb,\text{4}.\text{6)}\\ +\;\{ARF\;/\;MOSF\}[-\text{2}.\text{3549}WBC\\ +\;\text{2}.\text{7494(}WBC-{\text{8)}}_{+}-0.\text{4638(}WBC-{\text{11)}}_{+}] \end{array}$$

where:*Alb*: albumin; *Aresp*: APACHE III respiration score; *Bil*: bilirubin; *Cr*: Creatinine; *Na*: sodium; *PaO_2_*: partial pressure oxygen in arterial blood; *MeanBP*: mean arterial blood pressure; *WBC*: white blood cell count in thousands; *Temp*: temperature in Celsius; *HR*: heart rate per minute; *ARF*: Acute respiratory failure; *MOSF*: Multiple organ failure; *Cirrhosis*: Cirrhosis; *Coma*: Coma; *Lung*: Lung cancer; *ColonCancer*: Colon cancer; *COPD*: Chronic obstructive pulmonary disease; *CHF*: Congestive heart failure. Also:

$$\left\{disease\;group\right\}=\left\{\begin{array}{c} 1,\;\text{if}\;\text{patient}\;\text{in}\;\text{the}\;\text{disease}\;\text{group}\\ 0,\;\text{otherwise} \end{array}\right)$$

$${\left(x\right)}_{+}=\left\{\begin{array}{c} x,\;\text{if}\;x>0\\ 0,\;\text{otherwise} \end{array}\right)$$

*WBC *= 9, if *WBC *< 9 and {*disease group *} ≠ *ARF */*MOSF*

*WBC *= 40, if *WBC *> 40

*Cr *= 15, if Cr >15

The second step in implementing the SUPPORT model is to calculate the probability of survival for the individual patient based on equation A2 [22].

(A2) $$P\left\{T\geq t|\text{disease}\;\text{group}=i\right\}=S_{i}{\left(t\right)}^{e^{X_{\hat{b}}}}$$

where T: survival time in days; t: arbitrary time; S described in Table 1[22] and

**Table 1:** Values of Survival (S) as described in equation A2 for different disease types and varying survival times

t	S_ARF/MOSF_	S_COPD/CHP/Cirrhosis_	S_Coma_	S_Cancer_
**0**	0.994	0.998	0.993	0.993

**30**	0.691	0.889	0.630	0.578

**60**	0.601	0.837	0.609	0.407

**90**	0.562	0.800	0.581	0.264

**120**	0.532	0.772	0.569	0.190

**150**	0.508	0.751	0.551	0.135

**177**	0.493	0.733	0.545	0.108

$$\begin{array}{l} X_{\hat{b}}=-\text{3}.\text{652}+0.\text{8356\{}CHF\text{\}}+0.\text{9257\{}Cirrhosis\text{\}}\\ +\;0.\text{6287\{}LungCancer\text{\}}\pm \text{1}.\text{18}0\text{3\{}MOSFw\;/\;Malig\text{\}}\\ +\;0.0\text{1434}Scoma\pm 0.0\text{1935}Age+0.\text{2413}Cancer\\ -\;\text{1}.\text{863}{\left[Hday+\text{3}.\text{4}\right]}^{-}{}^{\text{1}}+0.0\text{8121}SPS\\ +\;Age[0.0\text{15261\{}COPD\;/\;CHF\;/\;Cirrhosis\text{\}}\\ +\;0.00\text{9}0\text{47\{}Coma\text{\}}-0.00\text{8294\{}Cancer\text{\}}]\\ +\;Age[-0.0\text{12498\{}CHF\text{\}}-0.00\text{4578\{}Cirrhosis\text{\}}\\ -\;0.00\text{1435\{}LungCancer\text{\}}\\ -\;0.0\text{13891\{}MOSFw\;/\;Malig\text{\}} \end{array}$$

where *Scoma*: SUPPORT coma score (0-100); *MOSFw*/*Malig*: Multiple organ failure with malignancies; *Hday*: day in hospital when qualified for study; *Cancer*: Cancer by comorbidity or primary disease category (0 = no; 1 = present; 2 = metastatic) [22].

### Derivation of the Expected Regret functions

As outlined in the Introduction, seriously and terminally ill patients may reap a number of benefits by the hospice program. Nevertheless, after enrollment into hospice, the patient (or the family, or the physician) may feel that this was a wrong decision, and subsequently may regret it. Similarly, the patient may feel regret for the treatment that he/she continues to receive because it is unnecessary, inappropriate, and/or harmful. Figure 3 represents our hospice decision tree in terms of regret from which we can compute the expected values of regret associated with each strategy as follows:

(A3) $$\begin{array}{c} ERg\left[Hosp\right]=\\ \left(1-p\right)*\left(U_{4}-U_{2}-H_{Rx}-H_{Hosp}\right) \end{array}$$

(A4) $$ERg\left[Rx\right]=p*\left(U_{1}-U_{3}+H_{Rx}-H_{Hosp}\right)$$

(A5) $$\begin{array}{c} ERg\left[SUPPORT\right]=p*TP*H_{te}\\ +\;\left(1-p\right)*FP\\ {}*\left(U_{4}-U_{2}-\left(H_{Rx}-H_{Hosp}\right)+H_{te}\right)\\ +p*FN*\left(U_{1}-U_{3}+\left(H_{Rx}-H_{Hosp}\right)+H_{te}\right)\\ +\left(1-p\right)*TN*H_{te} \end{array}$$

The variables *TP*, *FP*, *TN*, *FN *are related to the probabilities *P *(*p *≥ *P_t _*∩ *D *+), *P *(*p *≥ *P_t _*∩ *D *-), *P *(*p *<*P_t _*∩ *D *-) and *P *(*p *<*P_t _*∩ *D *+) respectively, and are estimated as follows:

• *P *(*p *≥ *P_t _*∩ *D *+) ≈ the number of patients who will die within 6 months and for whom the prognostic probability is greater than or equal to *P_t _*(with #TP = number of patients with true positive results, $P\left(p\geq P_{t}\cap D\;+\right)\approx \frac{\#TP}{n}$, where *n *is the total number of patients in the study).

• *P *(*p *≥ *P_t _*∩ *D *-) ≈the number of patients who will survive for longer than 6 months and for whom the prognostic probability is greater than or equal to *P_t _*(with #FP = number of patients with false positive results, $P(p\geq P_{t}\cap \;D-)\approx \frac{\#FP}{n})$.

• *P *(*p *<*P_t _*∩ *D *+) ≈the number of patients who will die within 6 months and for whom the prognostic probability is less than *P_t _*(with #FN = number of patients with false negative results, $P(p<P_{t}\cap \;D+)\approx \frac{\#FN}{n})$.

• *P *(*p *<*P_t _*∩ *D *-) ≈the number of patients who will survive for longer than 6 months and for whom the prognostic probability is less than *P_t _*(with #TN = number of patients with true negative results, $P(p<P_{t}\cap \;D-)\approx \frac{\#TN}{n})$.

To incorporate the effects of alternative treatments (e.g. treatment and hospice care) in equations A3-A5 we use the *Relative Risk Reduction *reported in literature for each strategy as follows:

(A6) $$\begin{array}{c} ERg\left[Hosp\right]=\left(1-p\right)*\left(1-RRR_{Hosp}\right)\\ {}*\;\left(U_{4}-U_{2}-H_{Rx}-H_{Hosp}\right) \end{array}$$

(A7) $$\begin{array}{c} ERg\left[Rx\right]=p*\left(1-RRR_{Rx}\right)\\ {}*\;\left(U_{1}-U_{3}+H_{Rx}-H_{Hosp}\right) \end{array}$$

(A8) $$\begin{array}{c} ERg\left[SUPPORT\right]=\\ p*\left(1-RRR_{Hosp}\right)*TP*H_{te}\\ +\left(1-p\right)*\left(1-RRR_{Hosp}\right)*FP\\ {}*\left(U_{4}-U_{2}-\left(H_{Rx}-H_{Hosp}\right)+H_{te}\right)\\ +p*\left(1-RRR_{Rx}\right)*FN\\ {}*\left(U_{1}-U_{3}+\left(H_{Rx}-H_{Hospe}\right)+H_{te}\right)\\ +\left(1-p\right)*\left(1-RRR_{Rx}\right)*TN*H_{te} \end{array}$$

Since *TP *+ *FN *= 1 and *FP *+ *TN *= 1, we have:

$$\begin{array}{c} p*TP+\left(1-p\right)*FP+p*FN\\ +\left(1-p\right)*TN=p+\left(1-p\right)=1 \end{array}$$

Therefore, equation A8 becomes:

(A9) $$\begin{array}{l} ERg[SUPPORT]=\\ (1-p*RRR_{Hosp}*TP-(1-p)*RRR_{Hosp}*FP\\ -p*RRR_{Rx}*FN-(1-p)*RRR_{Rx}*TN)*H_{te}\\ +(1-p)*(1-RRR_{Hosp})*FP\\ {}*\left(U_{4}-U_{2}-(H_{Rx}-H_{Hosp})\right)\\ +p*(1-RRR_{Rx})*FN\\ {}*\left(U_{1}-U_{3}+(H_{Rx}-H_{Hosp})\right) \end{array}$$

Scaling the equations A3, A4 and A9 with the quantity (*U*_1 _- *U*_3 _+ *H_Rx _*- *H_Hosp_*) and replacing the expression $\frac{U_{4}-U_{2}-\left(H_{Rx}-H_{Hosp}\right)}{U_{1}-U_{3}+H_{Rx}-H_{Hosp}}$ with $\frac{P_{t}}{1-P_{t}}$, we derive the final equations for the expected regret (equations 3, 4, and 5).

## Pre-publication history

The pre-publication history for this paper can be accessed here:

http://www.biomedcentral.com/1472-6947/11/77/prepub
